# Hiking Time Trial Performance in the Heat with Real-Time Observation of Heat Strain, Hydration Status and Fluid Intake Behavior

**DOI:** 10.3390/ijerph17114086

**Published:** 2020-06-08

**Authors:** Joshua D. Linsell, Emily C. Pelham, David M. Hondula, Floris C. Wardenaar

**Affiliations:** 1 College of Health Solutions, Arizona State University, Phoenix, AZ 85004, USA; jlinsell@asu.edu (J.D.L.); emilypelham@gmail.com (E.C.P.); 2 School of Geographical Sciences and Urban Planning, Arizona State University, Tempe, AZ 85281, USA; david.hondula@asu.edu

**Keywords:** exertional heat illness (EHI), heat strain, heat stress, mountain search and rescue, biometeorology, dehydration, dietary behavior, public health and safety

## Abstract

This study investigated the real-time progression of heat strain in mountain hikers during time trials (TT). Participants (*n* = 12; 7M/5F; age 21.6 ± 2.47) attempted to climb Tempe Butte (~1.1 mi) four times in HOT and MOD trials (wet bulb globe temperature 31.6 °C vs. 19.0 °C). Performance, physiological outcomes, and fluid intake behavior were measured in real-time. Hot conditions significantly impaired hiking TT performance by 11%, reduced aerobic capacity by 7%, increased peak rate of perceived exertion (RPE) by 19%, and elevated core temperature (T_c_) by 0.7 °C compared to MOD (all *p* < 0.03). Less-aerobically-fit participants were most negatively-affected by heat stress. Based on sweat lost, participants in HOT required 2.26 ± 0.91 L of fluids, brought 1.52 ± 0.83 L, and consumed 1.54 ± 0.49 L, losing an average of 1.1% ± 1.0%BM. Participants in MOD required 1.28 ± 0.39 L of fluids, brought 1.57 ± 1.09 L, and consumed 0.79 ± 0.57 L, losing an average of 1.0% ± 0.8%BM. Morning-after urine specific gravity (USG) values revealed 75% of hikers were hypohydrated (USG ≥ 1.020) after HOT; 67% after MOD. Heat stress impairs hiking TT performance while increasing RPE and T_c_. Fitter participants showed less performance and physiological impairment from heat stress. Although hikers in both conditions lost similar body weight, hikers were limited in HOT by fluid availability, whereas in MOD, fluid was available and dehydration was voluntary.

## 1. Introduction

Hiking remains the most popular activity among the nearly 200 million annual visitors to US National Forests [1]. Although most hiking is uneventful, hiking can be dangerous. Exertional heat illness (EHI) is a common cause of mountain rescues, especially in hot environments [2]. Exertional heat illness is a form of heat-related illness (HRI), which is caused by individual and/or environmental heat stress while performing physical activity. Such illnesses range in severity from heat edema, heat cramps, heat syncope, and heat exhaustion to life-threatening heat stroke [3]. Records from Grand Canyon National Park in Arizona, USA, show that HRI incidents made up 8.7% of total emergency medical service (EMS) incident responses (most of which involved hiking), with 25% of the cases being clinically dehydrated [2]. Further south in the sprawling Phoenix metropolitan area (mean elevation: 331 m; mean summer high temperature: 40.1 °C) of Arizona, mountain rescues are on the rise, reaching 255 incidents in 2019 [4]. Authorities in the Phoenix area report anecdotally that most of the rescues are either heat-related illnesses or musculoskeletal injuries [5].

Fortunately, EHI can be prevented, but in order to do so, scientists and safety officials must seek a thorough understanding of the mechanistic nature of EHI. Many environmental and individual factors contribute to whether or not heat stress results in heat strain or EHI. Most apparent is the notion that high ambient temperature (an environmental factor) can cause HRI, but other environmental variables such as relative humidity, radiative heat, and wind speed each affect how the individual experiences heat stress. Less understood is the role of individual factors contributing to heat stress, including acute variables such as body temperature, work rate (intensity of activity), duration of activity/exposure, hydration status, acute physical condition (e.g., fever), and the use of certain medications [3]. Chronic variables (involving adaptation/maladaptation over time) include: sweat rate, acclimatization status, cardiorespiratory fitness level (aerobic fitness or aerobic capacity), and certain chronic physical conditions [6]. Ultimately, it is the interaction between environmental and individual factors that causes EHI.

Many of the current rescue prevention programs in the USA utilize information gathered from retrospective rescue data [7], experiments in laboratory settings involving different activities (cycling or treadmill walking) [8], or post-hike surveys [9]. Currently, no studies have assessed real-time mountain hiking performance in the heat, despite the well-established notion that heat impairs submaximal aerobic performance before the onset of fatigue [8] and fatigue generally precedes EHI [10]. Additionally, no study has objectively assessed hiker preparedness relative to prevention program recommendations. Prevention programs such as the “Take a Hike–Do it Right” campaign in Arizona lack specificity, recommending hikers to “be honest” and “take responsibility” rather than quantifying adequate fluid amounts, appropriate fitness levels, or the importance of incremental exposure (acclimatization) for specific trails and environmental conditions [11]. There exists a great need for real-time observations on which to base current and future hiking safety recommendations for the prevention of EHI. It is the goal of this study to provide such evidence by continuously observing the progression of heat strain, hydration status, and food and fluid intake behaviors in mountain hikers.

## 2. Materials and Methods

### 2.1. Study Design

In this quasi experimental study participants served as their own control. The study activity was defined as hiking the Tempe Butte (“A-Mountain”; peak elevation 456 m) in Tempe, Arizona, four consecutive times, covering a distance of 901 m (0.56 mile) and prominence of 106 m (330 ft) in the summer (HOT) and fall (MOD) of 2018. Participants ingested a telemetric temperature capsule between 12 and 4 h prior to hiking, and a standardized breakfast (cereal and low-fat milk or soy milk: ~330 kcal; ~65 g carbohydrate; ~8 g protein; ~4 g fat) 2 h prior to hiking. Participants were instructed to abstain from caffeine and consume only water between their breakfast and the start of the hike (12:00 PM ± 1:00). Before the start of the hike, baseline metabolic rate, and body weight (in minimal dry clothing) were measured after delivering an “all-out” urine sample. Baseline core temperature, heart rate, and ratings of perceived exertion (RPE) were also recorded before the hike. During the hike, heart rate, breathing rate and core temperature were measured continuously. RPE was recorded at each passing of the “stations” set up at the base and summit of the butte. Participants’ start times were staggered by 1-min intervals to encourage self-selected pacing, and participants were instructed to hike at a sustainably “brisk” pace without running, simulating a submaximal aerobic time trial (TT) performance. Participants were able to withdraw voluntarily. During the hike, participants had ad libitum access to their own food and drink, which were weighed before and after the hike to assess consumption. Immediately after the hike, participants delivered a urine sample, and body weight was measured in the same set of dry clothes worn before the hike. Finally, a first morning urine sample was collected on the day after the hike.

### 2.2. Participants

Healthy adult participants between the ages of 18 and 40 were screened for eligibility using the following criteria: living ≥6 months in a hot-arid desert area (e.g., Phoenix Metropolitan area, AZ), not using tobacco or medication interfering with hydration status, not being pregnant or consuming >21 alcohol servings/week. The priori sample size of *n* = 12 was based on sweat rate difference reported by Baker et al. [12] using a calculated effect size based on a sweat rate of 0.89 L/h, with a probability error of 0.05, and power of 0.80. Together with the informed consent (study approved by the Western Institutional Review Board [WIRB]: 1187205), all participants signed a contraindication form for ingesting a telemetric temperature capsule.

### 2.3. Environmental Conditions

Temperature (°C), relative humidity (%), and the wet bulb globe temperature (WBGT) were measured in ten-minute intervals using 2 heat stress meters on tripods (Kestrel 5400, Nielsen-Kellerman, Boothwyn, Pennsylvania) placed along the hiking path at baseline and halfway up the mountain.

### 2.4. Procedures

#### 2.4.1. Hiking Performance and Energy Expenditure

Hike start, split (base and summit stations), and finish times were recorded (hh:mm:ss). Heart rate (HR), breathing rate (BR) and activity (3-axis accelerometer) were measured using an elastic chest strap activity monitor (Bioharness-3, Zephyr Technology, Annapolis, USA) at 1-s intervals. Work rate (absolute intensity expressed in metabolic equivalents, or METs) was estimated based upon a linear regression approach derived from the 3-axis accelerometer activity score (A), HR, and BR, using the following equation: METs = −1.1644 + (0.02947∙HR) + (5.8985∙A) + (0.03583∙BR) [13]. Total energy expenditure (TEE) was estimated by multiplying work rate by time (MET-h). Predicted aerobic capacity (aerobic fitness; VO_2max_) was calculated for each trial as the average absolute intensity (METs) divided by average relative intensity (%HR_max_). Relative intensity of exercise was expressed as a percent of age-predicted maximum heart rate (%HR_max_) based on the following equation: HR/(208–[0.7∙age]) [14].

#### 2.4.2. Ratings of Perceived Exertion (RPE)

Participants were asked to assess their RPE before beginning the hike and each time they arrived at a base or summit station. We used the Borg RPE scale [14], which ranges from 6 to 20. In this scale, 6 represents “no exertion at all” and 20 represents “maximal exertion”.

#### 2.4.3. Core Temperature

A portable telemetry system (CorTemp^TM^ system, HQ Inc., Palmetto, FL, USA) was used to measure core temperature (T_c_) at 10-s intervals. Baseline T_c_ measurements at our lab facility (3 consecutive measurements) were taken ~10 min pre-hike. After this, each base and summit station real-time core temperature was registered and individual peak core temperatures were identified after data was logged post-hike.

#### 2.4.4. Basal Metabolic Rate

A handheld metabolic analyzer (Breezing Co., Tempe, AZ, USA) [15] was used to measure oxygen consumption, carbon dioxide production, and the respiratory exchange ratio (RER; VCO_2_/VO_2_) of participants before the hike as described by the manufacturer. Basal metabolic rate was calculated from VO_2_ and VCO_2_ using the Weir equation [16].

#### 2.4.5. Food and Fluid Intake Behavior

Participants were instructed to bring their own food and drink in preparation for a ~4-mile mid-day hike in existing local weather conditions. All food and drink brought and consumed by participants were labelled, recorded and weighed within 0.1-g accuracy on a precision scale (Sartorius ENTRIS623-IS) pre- and post-consumption to calculate consumed weight to be included in the sweat rate calculation. Fluid needs were defined as being equal to total sweat loss (see Section 2.4.6) and were then compared to fluid brought in order to assess hiker preparedness.

#### 2.4.6. Sweat Rate, Changes in Body Mass, and Hydration Status

Sweat rate (mL/h) was calculated for each trial using the following equation: Sweat Rate (mL/h) = (pre-exercise body mass − post exercise body mass + food and fluid intake − urine output)/exercise duration [17]. Total sweat loss and relative change in body mass (%) during the hike were calculated using the pre- and post-hike measurements of body mass, food/fluid intake, and urine output.

Pre- and post-hike all-out urine samples were collected and measured within 0.1-g accuracy on a precision scale. Pre-hike and morning-after fresh urine samples used to assess chronic hydration status were stored at 5 °C and analyzed no later than 5 days after data collection. To determine chronic hydration status, urine specific gravity (USG) was analyzed using a 30-mL sample at 20 °C (PEN-refractometer, ATAGO, Tokyo, Japan) with USG ≥1.020 g/mL being indicative of hypohydration [17].

### 2.5. Statistics

Activity data were checked for anomalies. In rare cases of time-offset or missing activity data, the offset data were re-synced to performance times, and missing data were imputed with mean values. Results were analyzed using SPSS (version 25) and reported as mean ± standard deviation (sd) after confirming normal distribution using skewness, kurtosis, and visual inspection of histograms. Differences in measured dependent variable means between the hot and moderate hikes were analyzed using paired-samples *t*-tests. Differences in repeatedly measured variables within each environmental condition were analyzed using one-way repeated measures ANOVA. Difference scores were computed (hot–moderate) in order to compare between-groups effects with the one-way repeated measures ANOVA. In cases where the assumption of normal distribution was not met in the repeated measures data, the Greenhouse–Geisser corrected degrees of freedom were used to interpret F-statistics. Significance for all tests was set at *p* ≤ 0.05.

## 3. Results

### 3.1. Participants and Environment

Twelve hikers (seven male, five female; mean age 21.6 ± 2.47y) participated in the study. Participants hiked during HOT summertime conditions (WBGT 31.6 °C) and then again during MOD fall conditions (WBGT 19.0 °C). Four participants (hikers 1, 2, 3, and 4) did not finish the HOT hike–hikers 1 and 2 withdrew after the first climb (of four), hiker 3 withdrew after the third climb, and hiker 4 withdrew during the fourth and final climb. Hikers 1, 2, and 4 did not return for the hike under MOD conditions. As a result, nine participants (seven male, two female; mean age 22.1 ± 2.62y) returned in the fall to complete the study. Statistical comparisons between trials were based on these nine hikers who participated in both trials. Descriptive data for both HOT and MOD hikes can be found in Table 1.

### 3.2. Performance and Markers of Physiological Heat Stress

Hiking time trial performance was impaired by 11% in HOT compared to MOD conditions (*p* = 0.01). Summit station core temperatures were significantly higher (+0.51 °C) in HOT versus MOD (*p* = 0.001). The predicted aerobic capacity (VO_2max_) of participants was significantly lower (−7%) in the HOT trial versus MOD (*p* = 0.03). Individually, hikers 12 and 5 had the highest and second-highest predicted aerobic capacities in HOT. Hiker 11 was the least fit of the finishers and hiker 2 had the lowest predicted aerobic capacity of the entire group in HOT. No differences were found for estimated relative energy expenditure (*p* = 0.08) or estimated total energy expenditure (*p* = 0.41).

**Figure 1:**
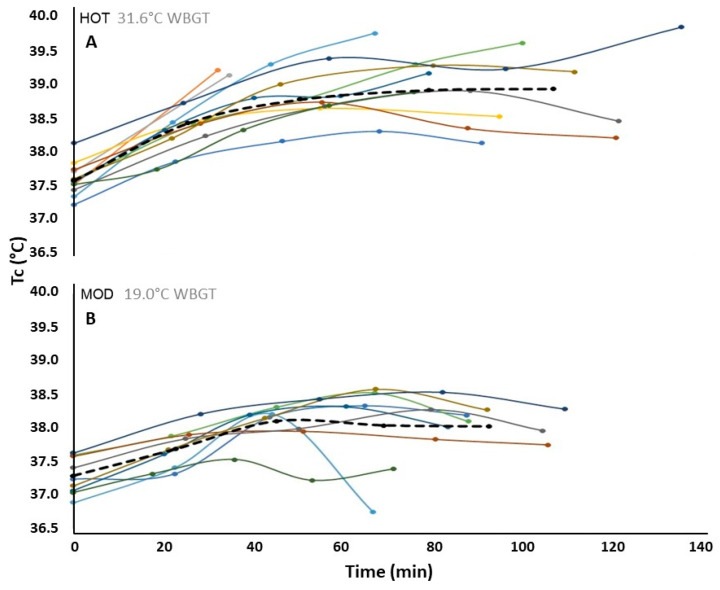
Individual (solid lines) and group average (dashed lines) core temperature (T_c_) versus (**A**) HOT and (**B**) MOD hiking split time.

Core temperatures rose progressively during the HOT trial from baseline (37.5 ± 0.28 °C) through climb 4 (38.9 ± 0.60 °C), but plateaued on the second climb in MOD at 38.1 ± 0.26 °C from a baseline of 37.3 ± 0.28 °C (Figure 1a,b). Baseline, average, and summit-station core temperature (T_c_) in finishers were all were higher in HOT (*p* < 0.02). Average hiking split times for climbs 1–4 (Figure 1a,b) were significantly higher in HOT (from 24.3 ± 3.89 min for climb 1 to 28.1 ± 7.47 min for climb 4) compared to MOD (finishers only; *p* = 0.01). In both HOT and MOD trials, hiking pace (split times for climbs 1–4) did not differ within subjects (*p* = 0.11, and *p* = 0.17, respectively) or within-subjects between trials (*p* = 0.21). Hiker 12 completed HOT in 76.6 min with the lowest first-climb and second-lowest average core temperature (37.7 and 38.1 °C, respectively). Hiker 5 had the lowest peak and average base station core temperatures in HOT (38.43 °C and 37.91 °C, respectively). Hiker 11 finished HOT in 136.7 min with the highest average and peak core temperatures of the group (39.0 and 39.82 °C, respectfully). The two hikers with the slowest split times for the first climb in HOT (hikers 1 and 2; 32.4 and 34.9 min, respectively) also achieved the highest core temperatures for that climb (39.18 and 39.11 °C, respectively) immediately before withdrawing from HOT.

**Figure 2:**
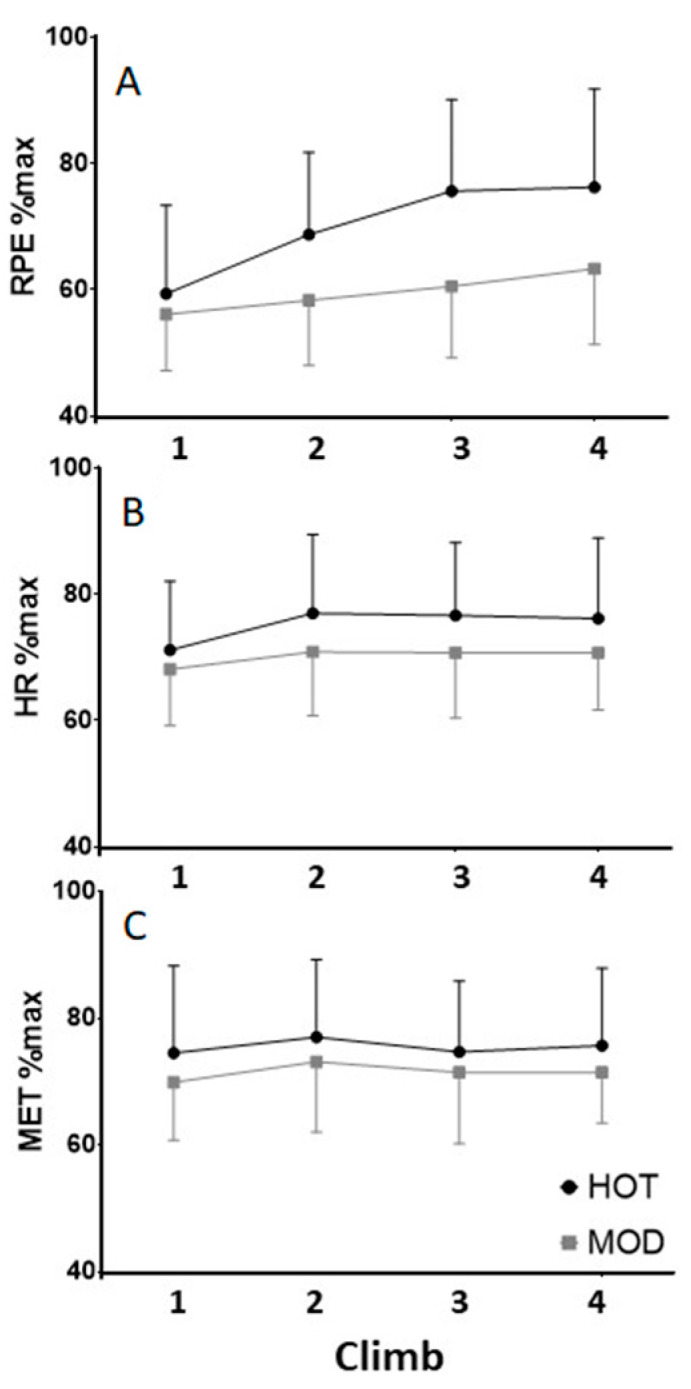
Percent of maximal values for (**A**) summit-station ratings of perceived exertion, (**B**) heart rate, and (**C**) work rate, during each climb.

Ratings of perceived exertion recorded at the summit of each climb (indexed in Figure 2a) increased rapidly in HOT from 11.9 ± 2.80 to 15.3 ± 3.11 and were significantly higher than in MOD (*p* = 0.01). Ratings in MOD ranged from 11.2 ± 1.79 to 12.7 ± 2.40 and were not significantly different between climbs. Relative intensity, measured by percent of age-predicted maximum heart rate, increased from 71.1 ± 11.0 to 76.1 ± 12.7 in HOT (*p* = 0.12) and from 69.1 ± 8.94 to 71.8 ± 9.12 in MOD (*p* = 0.02) (Figure 2b). However, no significant difference was found between trials (*p* = 0.32). Similarly, absolute exercise intensity (METs) failed to reveal any differences within HOT (from 5.92 ± 0.52 to 6.03 ± 0.72; *p* = 0.64), MOD (from 5.93 ± 0.55 to 6.06 ± 0.52; *p* = 0.24), or between trials (*p* = 0.93). Figure 2c displays relative intensity by depicting METs as a percentage of predicted aerobic capacity (VO_2max_).

### 3.3. Hydration Status

#### 3.3.1. Acute Changes in Hydration Status

The total amount of sweat lost was 2269 ± 910 mL for HOT and 1280 ± 390 mL for the MOD trial (*p* = 0.006). Sweat rates differed by 0.54 L/h (*p* = 0.010) based on group values of 1.4 ± 0.5 L/h for HOT and 0.8 ± 0.3 L/h for MOD conditions. Percentages of body weight loss were not found to be significantly different in the HOT and MOD trials (1.1% ± 1.0% and 1.0% ± 0.8%, respectively). Eight out of twelve participants (67%) exhibited body weight losses ≥1% in the HOT trial versus five out of nine (56%) during the MOD trial. The fastest and fittest participant (hiker 12) exhibited a body weight loss of 2% during HOT conditions, and two (hikers 10 and 12) lost over 2% bodyweight during MOD conditions.

There was a statistically significant difference (*p* < 0.001) in the amount of fluid consumed on the HOT and MOD hiking days with a mean difference of 753 ± 369 mL (HOT: 1541 ± 485 mL; MOD: 787 ± 565 mL). Urine excretion was significantly lower during HOT conditions (85.4 mL) vs. MOD conditions (232 mL), *p* < 0.001.

#### 3.3.2. Baseline Hydration Status before and Day after the Hike: Urine Specific Gravity

Hikers were euhydrated based on their pre-hike USG values. Urine specific gravity on HOT and MOD days was 1.016 ± 0.010 and 1.010 ± 0.008 respectively. However, 50% of hikers (6 of 12) arrived for the HOT hike with a USG value >1.020, where only 11% (1 of 9) arrived for the MOD hike with a USG value higher than 1.020. The morning-after USG values were 1.022 ± 0.007 for HOT and 1.019 ± 0.009 for the MOD condition. Both differed significantly from the hiking day before (*p* = 0.047 for HOT and *p* = 0.026 for MOD), resulting in 75% and 67% of participants with USG values >1.020, respectively.

### 3.4. Food and Fluid Intake Behaviors in Relation to Replacing Fluid Loss

**Figure 3:**
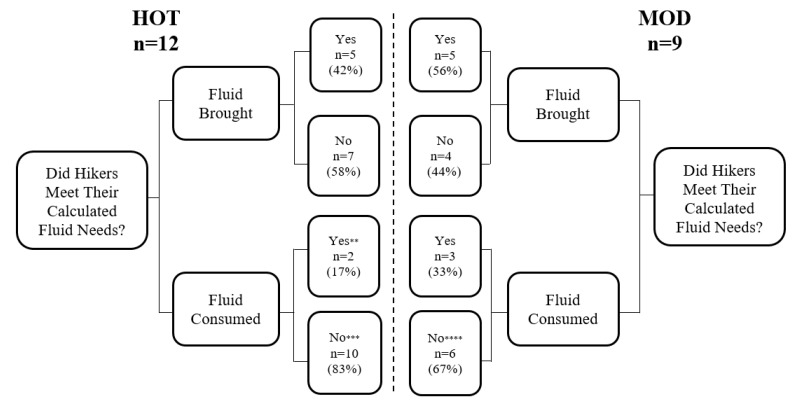
Categorization of hikers’ fluid behavior in HOT and MOD. ** One hiker met fluid needs by using “rescue water” (RW); *** Five hikers used RW and still did not meet their needs; **** One hiker used RW and still did not meet personal fluid needs.

The average amount of food consumed during HOT (33.2 ± 79.7 g) and MOD (0.0 ± 0.0 g) was not significantly different (*p* = 0.248). 58% of hikers did not bring enough fluids to replace their sweat loss in the HOT trial, and 44% did not bring enough for the MOD trial (Figure 3). During both trials, hikers did not consume enough fluids to make up for their personal sweat losses. This was true for 83% of participants in HOT conditions and 67% for MOD.

**Figure 4:**
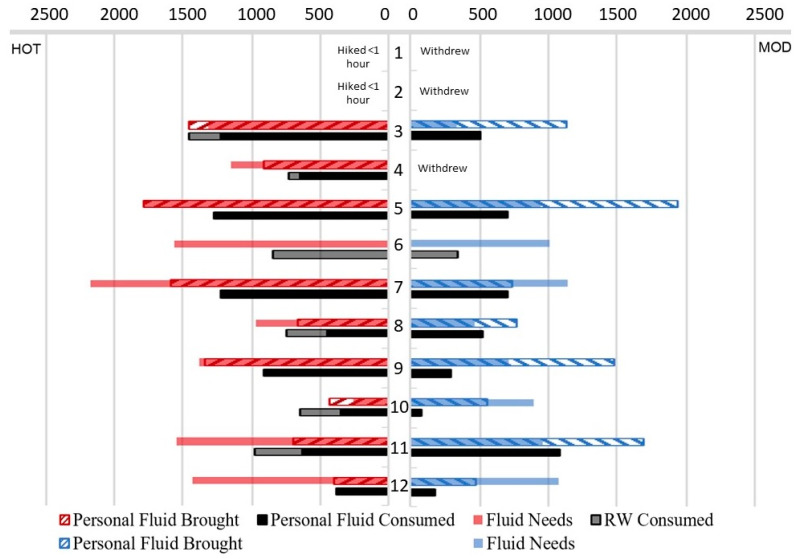
Fluid needs (sweat loss) in mL versus fluid brought and consumed in HOT and MOD. Note: Data of participants 1 and 2 is not included as they completed only one of four climbs.

During HOT conditions, only three hikers (hikers 3, 5, and 10) matched their fluid brought to their fluid lost (fluid needs) (Figure 4). During MOD conditions, five out of nine brought more fluid than they needed. “Rescue water” was offered on request at the base station to participants that ran out of personal fluids. If “rescue water” had not been offered during the HOT day, only one participant (hiker 10) would have been able to replace their personal fluid loss (not including hiker 3, who did not finish the HOT hike). Hiker 10 was also the only participant to consume more than needed, gaining body weight during HOT. During MOD, relatively more fluid was brought in relation to the fluid needs, and “rescue water” was used only by the one hiker that did not bring any fluids for either testing day.

## 4. Discussion

Environmental heat stress (T_dry_ = 40.4 ± 2.50 °C; WBGT = 31.6 ± 2.10 °C) seems to impair mountain-hiking time trial (TT) performance, increase core temperature and heart rate, and influence hydration status and drinking behavior in comparison to a moderate environment (T_dry_ = 22.9 ± 1.60 °C; WBGT = 19.0 ± 0.74 °C).

Hiking time trial performance was indeed negatively impacted by environmental heat stress as shown by the 11% time difference between HOT and MOD trials. These results add mountain-hiking to the list of other time trial performance activities (cycling, running, and walking) impaired by heat stress by an average of 13% [8]. The estimated 7% reduction in aerobic capacity between HOT and MOD condition in the present study was slightly lower than the 11% average decrease in VO_2max_ described by Nybo et al. (2014) [8]. These reductions in performance time and aerobic capacity are important indicators of the heat strain experienced by individuals, as performance decrements are usually seen well before the occurrence of exertional heat illness [10].

Our study was the first to measure core temperature (T_c_; a common measure of heat strain) in mountain hikers during time trials in hot and moderate environments. We found a 0.7 °C elevation in mean core temperature of hikers in HOT versus MOD time trials. In comparison, a 2014 review [8] of 15 studies on submaximal aerobic performance found that environmental heat stress conditions resulted in a 0.4 °C higher mean core temperature compared to moderate conditions. The amplified T_c_ response in our study may be due to differences in environment, as our “hot” condition was hotter (T_dry_ = 40.4 °C) than that of any of the 15 studies reviewed (25–40 °C), although WBGT was not reported in those studies. Some of the studies included in the review were time-to-exhaustion (TTE) performances, which restricts subjects to constant work rates between trials, therefore limiting their applicability to self-paced performance. Additionally, none of those 15 studies examined outdoor mountain hiking performance. –Instead, many of them examined treadmill running, walking, or cycling performance in controlled lab environments. The results of our study demonstrate the real-world, grueling nature of summertime hiking in the desert southwest.

The early withdrawal of the slowest two participants (hikers 1 and 2 (also the least fit)) described in this study coincided with their rapid rise in core temperature relative to the group, quickly attaining “critical” core temperatures (tolerable upper-limit) commonly observed in persons with lower aerobic fitness [10]. The slowest and least-fit participant to complete all four climbs in HOT (hiker 11; time = 137 min) also had the highest average T_c_ and attained the highest peak T_c_ of the group just after finishing, coincident with the onset of minor heat cramps. Conversely, the fittest participant in HOT (hiker 12) had the fastest completion time, lowest first-climb T_c_, and second-lowest average T_c_ of the group. The second-fittest participant (hiker 5) had the lowest peak and average base station core temperatures in HOT. Environmental heat stress was shown to significantly raise core temperatures of mountain hikers (especially the slower and lesser-fit) compared to moderate conditions, and participants’ voluntary withdrawal from the HOT time trial coincided with the tolerable upper-limit core temperatures commonly observed in the literature [10].

Average summit-station ratings of perceived exertion were 18% higher in the HOT versus MOD trials, and this represented the most significant result among our study’s measures of exercise intensity. Both measures of relative exercise intensity (percent HR_max_, and percentage of predicted aerobic capacity [VO_2max_]) and absolute intensity (work rate per kilogram; METs) were higher in HOT, but none of them achieved statistical significance. We had hypothesized that the increased cardiovascular strain associated with heat stress would result in higher absolute exercise intensity in the heat, but this was under the assumption of a similar hiking pace and aerobic capacity (VO_2max_) between trials. In reality, both hiking pace (time trial performance) and aerobic capacity appeared to be impaired by environmental heat stress as described above. Hikers may have adjusted their pacing (hiking speed) to hike at a comparable absolute intensity (METs), relative intensity (heart rate or METs relative to maximal values), or both, between trials. Although RPE is subjective, ratings at the summit of each climb were our best and earliest indicators of heat strain in mountain hikers.

During hiking, participants had greater sweat losses (fluid needs) and higher sweat rates in HOT than in MOD. Only one participant (hiker 12) attained a hypohydrated state (≥2% loss of body mass) in the HOT trial, but he was also the fittest and fastest hiker of the group. Hikers 3 (non-finisher) and 10 (finisher) consumed more fluid than they lost in HOT, putting them at risk for exercise-associated hyponatremia (EAH) [18]. Having one finisher dehydrate (hiker 12) and one overhydrate (hiker 10) is comparable to the aforementioned 2013 study by Noe et al. [2] where the ratio of clinical hypohydration to hyponatremia HRI incidents was 1 to 0.76. Other hikers in our HOT condition may have been at risk for EAH due to high hypotonic fluid consumption (≥1000 mL/h) or by failing to replenish appropriate amounts of electrolytes via food or sport drinks [19]. Our study did not seek to test for EAH, and the duration of the hike was probably insufficient to elicit symptoms, so these specific results should be interpreted with caution. Even though sweat rates were significantly higher in HOT, the individual variability in sweating rate found in our study and others [12] makes it difficult to generalize for public recommendations of fluid needs. Our relatively ambiguous results regarding hydration outcomes in the heat is congruent with the latest evidence for exertional heat stress (EHS) mitigation strategies, which places fluid ingestion last below pre-exercise cooling, heat acclimatization, and aerobic fitness in terms of its ability to favorably alter core temperature during EHS [20].

In the MOD condition, hikers 10 and 12 became hypohydrated, but the group average loss of body weight did not differ between trials. Given that the group did not experience clinical hypohydration before or during hiking in either condition, we submit that if starting euhydrated and given free access to water, severe dehydration during a ~1.75 h hike at ~40 °C is not to be expected in healthy hikers. Fluid consumption was inadequate during both trials, but fluid availability was the limiting factor in HOT, whereas fluid intake behavior limited fluid replacement in MOD. Post-activity rehydration (as measured by morning-after urine samples) deserves attention as well. On average, hikers reached higher urine concentrations after the HOT trial, indicating hypohydration, but not after the MOD trial. Although fluid replacement after hiking cannot improve the hiking performance itself, inadequate fluid replacement may attenuate the adaptation response to heat stress and negate the potential benefit for future heat exposures, especially in older adults [17].

## 5. Conclusions

Environmental heat stress appears to impair mountain hiking time trial performance, while increasing core temperatures and ratings of perceived exertion in mountain hikers, compared to moderate conditions. Fitter participants showed less performance and physiological impairment from heat stress. Fluid consumption was higher in the HOT condition, but body weight loss averaged 1% in both conditions. Although hikers in both conditions lost similar body weight, hikers were limited in hot conditions by fluid availability, whereas in moderate conditions, fluid was available and dehydration was voluntary. Therefore, we suggest that public health officials focus on the importance of fluid availability when the amount of fluid brought may limit the ability to maintain fluid balance in the heat (e.g., when summer hikes exceed roughly 1.5 h in duration). Additionally, based on the evidence, safety recommendations for hikers should emphasize aerobic fitness and gradual heat exposure (acclimatization) to foster familiarity with personal physiological responses to exertional heat stress, and to mitigate the occurrence of EHI and mountain rescues.

## Figures and Tables

**Table 1:** Descriptive data for HOT and MOD hikes.

	HOT	MOD	*p*
	mean ± sd	mean ± sd	
Subjects (M/F)	*n* = 7/5	*n* = 7/2	--
Subject Age (y)	21.6 ± 2.47	22.1 ± 2.62	--
Body Mass (pre-hike; kg)	69.8 ± 13.1	74.6 ± 11.9	--
Finishers/Total Hikers	*n* = 8/12	*n* = 9/9	--
Wet Bulb Globe Temperature (WBGT; °C)	31.6 ± 2.10	19.0 ± 0.74	--
Ambient Dry Bulb Temperature (T_dry_; °C)	40.4 ± 2.50	22.9 ± 1.60	--
Relative Humidity (%)	21.4 ± 2.92	18.2 ± 1.38	--
Total Participant Hiking Time (min)	105 ± 21.7	93.9 ± 13.1	0.013
Summit Core Temp. (°C)	38.5 ± 0.36	38.0 ± 0.30	0.001
Predicted Aerobic Capacity (fitness; METs)	7.99 ± 0.85	8.51 ± 0.88	0.026
Relative Energy Expenditure (MET-h)	10.1 ± 2.45	9.03 ± 1.50	0.082
Total Energy Expenditure (kcal)	727 ± 302	684 ± 223	0.409
